# Transcription factor 25 modulates gametocytogenesis and ribosome biogenesis in the malaria parasite *Plasmodium falciparum*

**DOI:** 10.3389/fcimb.2025.1652542

**Published:** 2025-10-24

**Authors:** Jing Wu, Zuping Zhang, Chandara Ngim, Wenyu Yang, Peiyi Li, Fei Wang, Jingru Ye, Bo Li, Bin Tian, Qingfeng Zhang, Xiaomin Shang

**Affiliations:** ^1^ Department of Parasitology, Xiangya School of Basic Medicine, Central South University, Changsha, China; ^2^ Laboratory of Molecular Parasitology, State Key Laboratory of Cardiology and Research Center for Translational Medicine, Shanghai East Hospital, School of Medicine, Tongji University, Shanghai, China; ^3^ Hunan Provincial Key Lab of Immunology and Transmission Control on Schistosomiasis (The Third People’s Hospital of Hunan Province), Yueyang, China; ^4^ Clinical Laboratory Department, Changsha Municipal Center for Disease Control and Prevention, Changsha, China

**Keywords:** *Plasmodium falciparum*, gametocytogenesis, transcription factor 25, rRNA, ribosome biogenesis

## Abstract

**Introduction:**

*Plasmodium falciparum*, the causative agent of severe malaria, predominantly reproduces through asexual stages within human red blood cells, with a small subset differentiating into transmissible gametocytes. TCF25 is recognized in other eukaryotes as a protein with dual roles: a transcriptional regulator and a key component of the Ribosome-associated Quality Control (RQC) complex. Nevertheless, the precise biological function of TCF25 in *Plasmodium* spp. remains inadequately elucidated.

**Methods:**

To investigate the function of TCF25, we created a *tcf25* knockout (*tcf25_ko*) parasite strain and conducted comparative transcriptomic analysis during the ring and schizont stages. Gametocyte induction experiments were performed to investigate the impact of *tcf25* deletion on gametocyte development. Chromatin immunoprecipitation sequencing (ChIP-seq) was utilized to delineate the genome-wide binding profiles in schizont-stage parasites. Additionally, RT–qPCR was used to quantify changes in rRNA expression levels after *tcf25* knockout.

**Results:**

Transcriptomic analysis of *tcf25_ko* parasites indicated substantial dysregulation, with 168 genes downregulated and 24 genes upregulated during the ring stage, and 53 genes downregulated and 4 genes upregulated during the schizont stage. ChIP-seq analysis identified 44 high-confidence TCF25-binding target genes, which notably included the rDNA. Furthermore, TCF25 deficiency resulted in upregulated rRNA expression, particularly affecting 28S rRNA, a core component of the 60S ribosomal subunit.

**Discussion:**

This study identifies TCF25 as a key regulator of various biological processes in *P. falciparum*. It is shown that TCF25 plays a crucial role in gametocytogenesis by influencing the *ap2-g* pathway. Additionally, a novel function of TCF25 in ribosomal biogenesis is uncovered, wherein it directly controls A-type rRNAs expression and ribosomal subunit homeostasis. These discoveries offer fresh perspectives on the molecular mechanisms that oversee transmission and ribosome biogenesis in malaria parasites.

## Introduction

Malaria, caused predominantly by *Plasmodium falciparum*, remains a significant global health concern, responsible for the majority of severe cases and fatalities. Recent data show an estimated 263 million clinical cases and 597,000 deaths in 2023, mainly attributed to cerebral malaria, the most fatal form of *P. falciparum* infection (World Health Organisation, 2024). The complex life cycle of *P. falciparum* involves alternating between human and mosquito hosts, creating crucial opportunities for interventions. Female Anopheles mosquitoes become infected by ingesting blood meals containing mature sexual-stage gametocytes. Inside the mosquito midgut, these gametocytes undergo sexual reproduction, developing into sporozoites capable of initiating new human infections, thereby completing the transmission cycle. Within human erythrocytes, the majority of parasites undergo multiple rounds of asexual replication, while a small percentage commit to gametocytogenesis. This subset follows a 10 to 12-day developmental process, culminating in the production of sexually mature forms capable of transmission (Karunajeewa and Mueller, 2016; Meibalan and Marti, 2017; Josling et al., 2018; Ngotho et al., 2019; Birkholtz et al., 2022; Cui and Kim, 2024).

This complex life cycle of the malaria parasite is coordinated by precisely controlled gene expression patterns that guide its developmental progression. Recent advances have identified AP2-G as the master transcriptional regulator controlling this developmental switch (Kafsack et al., 2014; Sinha et al., 2014). Forced AP2-G expression suffices to drive high-efficiency sexual conversion, demonstrating its central role in initiating the gametocyte differentiation program (Martins et al., 2017; Llorà-Batlle et al., 2020). AP2-G initiates the gametocytogenesis program through autoregulation and by regulating genes associated with early gametocyte development, a process involving the coordinated action of numerous proteins (Josling et al., 2020). Among these, proteins such as Pfg14-748, a target gene of AP2-G, exhibit expression patterns suggestive of its involvement in the transition from asexual to sexual development. Pfg14–748 is initially expressed in late-stage schizonts prior to the release of merozoites destined for gametocytogenesis, remaining within the parasitophorous vacuole throughout gametocyte development. And it is necessary for gametocyte maturation (Eksi et al., 2005; Silvestrini et al., 2010).

Transcription factors in *Plasmodium* are inadequately characterized, with the basic helix-loop-helix (bHLH) family emerging as a key regulator of eukaryotic gene expression, governing various biological processes from plant development to stress responses (Wei and Chen, 2018; Ke et al., 2020). A defining feature of bHLH proteins is their integration into complex regulatory networks fine-tuned by post-translational modifications (PTMs). Phosphorylation, ubiquitination, and SUMOylation dynamically modulate bHLH stability, subcellular localization, and DNA-binding affinity, thereby enabling precise control of transcriptional programs (De Masi et al., 2011; Selote et al., 2015; Gratz et al., 2019; Bernula et al., 2021; Srivastava et al., 2022). Beyond plants, bHLH factors exhibit conserved regulatory mechanisms with significant pathophysiological implications (Cai et al., 2006; Steen and Lindholm, 2008). Mammalian NULP1, also known as TCF25, acts as a suppressor of NFAT3-mediated transcriptional activity in cardiac hypertrophy, independently of calcineurin. This unexpected repressive function holds promise for therapeutic interventions (Zhang et al., 2020). Notably, the presence of a TCF25 homolog in *P. falciparum* prompts inquiries into its involvement in the parasite’s gene regulatory networks. In various eukaryotes, TCF25, known as Rqc1 in yeast, plays a pivotal role in the RQC complex. This complex serves as a surveillance mechanism that targets and eliminates defective polypeptides resulting from translational errors (Brandman et al., 2012; Defenouillère et al., 2016; Verma et al., 2018; Zurita Rendón et al., 2018; Barros et al., 2021). TCF25 facilitates K48-linked ubiquitination, thereby tagging faulty nascent chains for proteasomal degradation, a critical process for maintaining proteome integrity (Abaeva et al., 2025). Given the essentiality of protein homeostasis during *Plasmodium*’s complex life cycle, exploring whether the parasite’s TCF25 homolog governs RQC could unveil novel translational regulatory mechanisms. Understanding the transcriptional regulation and functional interactions of TCF25 in *P. falciparum* could offer valuable insights into how the parasite manages protein synthesis and degradation during its developmental stages.

In this study, we investigate the role of TCF25 during *P. falciparum* blood-stage development. Our findings demonstrate that TCF25 plays an essential role in gametocyte development and ribosome biogenesis. Disruption of the *tcf25* gene does not impact asexual replication but significantly hinders the rate of sexual conversion. By employing integrated transcriptomic profiling and ChIP-seq analyses, we have pinpointed numerous target genes directly regulated by TCF25. Our findings demonstrate that TCF25 plays an important role in gametocytogenesis through modulating the *ap2-g* pathway. Importantly, chromatin profiling and transcriptomic analyses identified TCF25 as a potential regulator of rDNA (encoding ribosomal RNA (rRNA)) gene clusters on specific chromosomes, with knockout studies demonstrating significant upregulation of these target transcripts. These findings demonstrate that TCF25 is essential in governing gametocytogenesis and ribosome biogenesis in *P. falciparum*.

## Materials and methods

### Parasite culture

The *Plasmodium falciparum* 3D7 strain was maintained in continuous culture using fresh human O+ erythrocytes at 2% hematocrit in complete RPMI 1640 medium supplemented with 0.5% Albumax II, 0.2% sodium bicarbonate, 25 mM HEPES, and 50 μg/mL hypoxanthine. Cultures were incubated at 37°C under controlled atmospheric conditions (5% O_2_, 5% CO_2_, and 90% N_2_), with medium replacement every 48 hours (Lu et al., 2021). For parasite synchronization, early ring-stage cultures were treated with 5% sorbitol at 37°C for 15 minutes to lyse mature-stage parasites, followed by washing with complete medium. Late-stage schizonts were isolated using a Percoll-sorbitol gradient (40% and 70% layers prepared in RPMI medium) by centrifugation for 20 minutes at 37°C without brake application. The schizont-enriched interface was carefully collected and washed with complete medium before reinvasion.

### Construction of transgenic strains

The plasmid construction was performed as previously described. Based on the pL6CS plasmid, we designed specific sgRNA sequences targeting the *tcf25* locus, and homology arms (approximately 1 kb each) were amplified by PCR from genomic DNA. Site-directed mutagenesis or HA-tag insertion was performed through overlap extension PCR, with the modified sequence flanked by the homologous arms. The final constructs were cloned into the pL6CS vector and Sanger sequencing prior to maxiprep purification. For transfection, 100 μg of the constructed plasmid and 100 μg of pUF1-Cas9 plasmid (dissolved in 150 μL of water) were co-transfected into fresh erythrocytes via electroporation. Transfected erythrocytes were immediately infected with Percoll-enriched schizont-stage parasites. The cultures were maintained under standard conditions until parasitemia reached 8-12%, typically after 1–2 replication cycles. Positive selection was initiated using WR99210 and DSM1, with drug pressure maintained for 3–4 weeks. Following morphological confirmation of normally developing parasites by Giemsa-stained thin blood smear microscopy, genomic DNA was isolated using the TIANamp Genomic DNA Kit (DP304; TIANGEN) according to the manufacturer’s protocol. Successful modification was initially screened by PCR, followed by agarose gel electrophoresis. PCR products of expected size were gel-purified and confirmed by Sanger sequencing, ensuring the absence of unintended mutations. This process successfully generated endogenous mutant parasite strains in the *tcf25* gene. The primers used in this study are listed in **Supplementary Table S1**.

### Growth curve analysis

Wild-type (WT) and *tcf25* gene knockout parasite strains were cultured *in vitro*. Following strict synchronization, the parasite growth window was restricted to 6 hours. At the trophozoite stage, initial parasitemia was adjusted to 0.1%, with parasites divided into WT and *tcf25* gene knockout groups. Cultures were maintained for four life cycles, and parasitemia was determined by microscopic examination during each trophozoite stage. A growth curve was generated and analyzed using GraphPad Prism 9.0.0.

### Gametocyte induction and analysis

Synchronous cultures of WT and *tcf25_ko* parasites were established at 2% initial parasitemia at 8–16 hours. After re-invasion, ring-stage parasitemia was determined by Giemsa-stained thin blood smears (parasitemia 1). Then, parasites were treated with 50 mM N-acetyl-D-glucosamine (GlcNAc; A3286, Sigma-Aldrich) in complete RPMI 1640 medium for 4 consecutive days at the next cycle to selectively eliminate asexual stage parasites while allowing gametocyte development. Gametocytemia was assessed by morphological identification in Giemsa-stained smears (parasitemia 2). The sexual conversion rate was calculated using the formula: Sexual conversion rate (%) = parasitemia 2/parasitemia 1. Each experiment included three biological replicates.

### Fluorescence microscopy

The *tcf25*-HA-tagged strain was maintained *in vitro*. Synchronization was achieved through sorbitol treatment at the ring stage. For immunofluorescence assays, parasites were harvested at different stages and immediately fixed in ice-cold 4% paraformaldehyde at 4°C. Fixed parasites were adhered to glass slides, permeabilized with 0.1% Triton X-100 in PBS for 5 min, and blocked with 1% BSA for 1 hour at room temperature. Immunostaining was performed using rabbit anti-HA multiclonal antibody (1:500 dilution; Cat#HA721750, HUABIO) followed by iFluor™ 488 Conjugated Goat anti-rabbit secondary antibody (1:500 dilution; Cat# HA1121, HUABIO), with each incubation for 1 hour at room temperature. Between incubations, slides were washed three times with PBS. For nuclear visualization, samples were stained with DAPI, then fluorescence images were acquired using a laser scanning confocal microscope (STELLARIS 5, Leica Microsystems). Image processing and analysis were performed using ImageJ software.

### Western blot

Western blotting was performed as previously described with modifications (Shang et al., 2022). Briefly, synchronized schizont-stage parasites were collected, and total protein was extracted by boiling at 100°C for 5 min. Proteins were separated on 8% SDS-polyacrylamide gels and transferred to a PVDF membrane. The membrane was blocked with 5% non-fat milk in PBST for 1 hour at room temperature, followed by incubation with antibodies overnight at 4°C. After three washes with PBST, the membrane was incubated with HRP-conjugated secondary antibody (1:5000 dilution; Cat#HA1001, HUABIO) for 1 hour at room temperature. Protein bands were detected using enhanced chemiluminescence (ECL) reagent and visualized using a ChemiDoc™ XRS+ imaging system with Image Lab™ Software (Bio-Rad).

### RNA-seq library preparation and high-throughput sequencing

Parasite cultures of WT and *tcf25_ko* strains were synchronized using our established growth assay protocol. Briefly, schizont-stage parasites were enriched by Percoll gradient centrifugation followed by ring-stage synchronization to achieve a narrow 6-hour growth window. After re-invasion, parasites were harvested at two developmental stages: ring (10–15 hpi) and schizont (40–45 hpi). Two biological replicates were used in this experiment. Then, parasite pellets were homogenized in TRIzol reagent (Transgen) and stored at -80°C. Total RNA was isolated using the TransZol Up Plus RNA kit (ER501) according to the manufacturer’s protocol. For RNA-seq library construction, 500 ng of high-quality total RNA per sample was used for the VAHTS Universal V10 RNA-seq Library Prep Kit (Vazyme, NR616). Briefly, poly(A)+ RNA was enriched using magnetic beads, followed by fragmentation and first-strand cDNA synthesis. After second-strand synthesis and adapter ligation, libraries were amplified with 14 cycles of PCR. Final library quality was assessed by Qubit 2.0 fluorometer and Agilent 2100 system. Paired-end sequencing was performed on an Illumina NovaSeq 6000 platform at BMK Biotechnology Co., Ltd (Beijing, China).

### RNA-seq data analysis

RNA-seq raw data were quality-trimmed using Trimgalore (v0.6.6) with default parameters, removing adapter sequences and low-quality bases. The cleaned reads were then aligned to the *Plasmodium falciparum* reference genome (PlasmoDB-v64) using HISAT2 (v2.2.1). Resulting SAM files were converted to BAM format and sorted using Samtools (v1.12). PCR duplicates were marked and removed using Picard tools (v2.26.0), followed by the generation of read count matrices with featureCounts (v2.0.1) against gene annotations. Read counts were analyzed using DESeq2 (v1.46.0) in R (v4.4.2). Approximately 20 million reads per sample were retained for downstream analysis. Genes with low counts were filtered prior to analysis. Differential expression was determined by applying thresholds of |log_2_ fold change| > 1 and *p*-value < 0.05.

### RNA extraction and RT-qPCR

Parasite cultures of WT and *tcf25_ko* strains were strictly synchronized as previously described. Ring-stage samples at approximately 10–15 hpi were collected from two independent clones. Total RNA was extracted using the manufacturer’s protocol. 500 ng of total RNA was reverse transcribed into cDNA using HiScript III RT SuperMix for qPCR (Vazyme, R323). RT-qPCR was performed with the following thermal cycling program: 95°C for 30 s, followed by 40 cycles of 95°C for 5 s, 54°C for 20 s, 56°C for 7 s, 59°C for 7 s, and 62°C for 27 s. The *seryl-tRNA synthetase* (PF3D7_0717700) gene was used as an internal reference for normalization. Relative gene expression levels were calculated using the 2^-ΔΔCt method. All samples were analyzed in triplicate using three biological replicates. The primers used are listed in **Supplementary Table S1**.

### Chromatin immunoprecipitations and high-throughput sequencing

Synchronous schizont-stage parasites (approximately 1×10^9 parasites) were cross-linked with formaldehyde and quenched with 0.125 M glycine. Erythrocytes were lysed using 0.15% saponin in PBS, followed by three washes with PBS. The parasite pellet was resuspended in lysis buffer and mechanically disrupted using a Dounce homogenizer. Chromatin was sheared to an average size of 100–500 bp using a sonicator. The sheared chromatin was centrifuged to remove debris. Then the supernatant was incubated with Protein A/G magnetic beads for 2 hours at 4°C with rotation. After magnetic separation, a small aliquot of the supernatant was reserved as an input control and stored at -80°C. The remaining chromatin was incubated overnight at 4°C with rotation in the presence of anti-HA antibody (2 μg) and Protein A/G magnetic beads (25 μL/mL). Then the immune complexes were washed sequentially with: low salt wash buffer, high salt wash buffer, LiCl wash buffer, and TE buffer. Finally, chromatin was eluted with the elution buffer by rotation for 30 min at room temperature. Cross-links were reversed by adding NaCl to 200 mM final concentration and incubating overnight at 45 °C. Samples were then treated with RNase A and Proteinase K. DNA was purified using the PCR Purification Kit. Libraries were prepared with the VAHTS Universal DNA Library Prep Kit for Illumina V4 (Vazyme) following the manufacturer’s instructions and sequenced on the Illumina NovaSeq 6000 platform (150 bp paired-end) at BMK Biotechnology. We used technical replicates in this experiment.

### ChIP-seq data analysis

Raw sequencing reads were quality trimmed using TrimGalore as described. The cleaned paired-end reads were aligned to the genome using Bowtie2 (v2.4.4). Resulting SAM files were converted to sorted BAM format using SAMtools (v1.15). Significant binding sites were identified by performing peak calling on two biological replicates using MACS2 (v2.2.7.1) with a threshold of q-value < 0.05. Peaks present in both replicates (overlap ≥ 60%) and with a fold enrichment greater than 1.8 were selected using Bedtools intersect (v2.31.1). These high-confidence peaks were subsequently annotated as target genes. Read coverage was normalized and visualized using bamCoverage to generate bigWig files with RPKM normalization. And Integrative Genomics Viewer was used for manual inspection of specific loci. All statistical analyses and visualizations were performed in RStudio (v4.4.2) using ggplot2 (v3.5.2) and ChIPseeker (v1.30.3) packages.

### GO enrichment analysis

Significantly differentially expressed genes were functionally annotated, and Gene Ontology (GO) enrichment analysis was performed in the PlasmoDB website (https://plasmodb.org/plasmo/app), with significance determined by the hypergeometric test (p < 0.05). All statistical analyses and visualizations were generated using ggplot2 (v3.5.2) in RStudio.

## Results

### TCF25 is not required for asexual blood-stage replication

**Figure 1:**
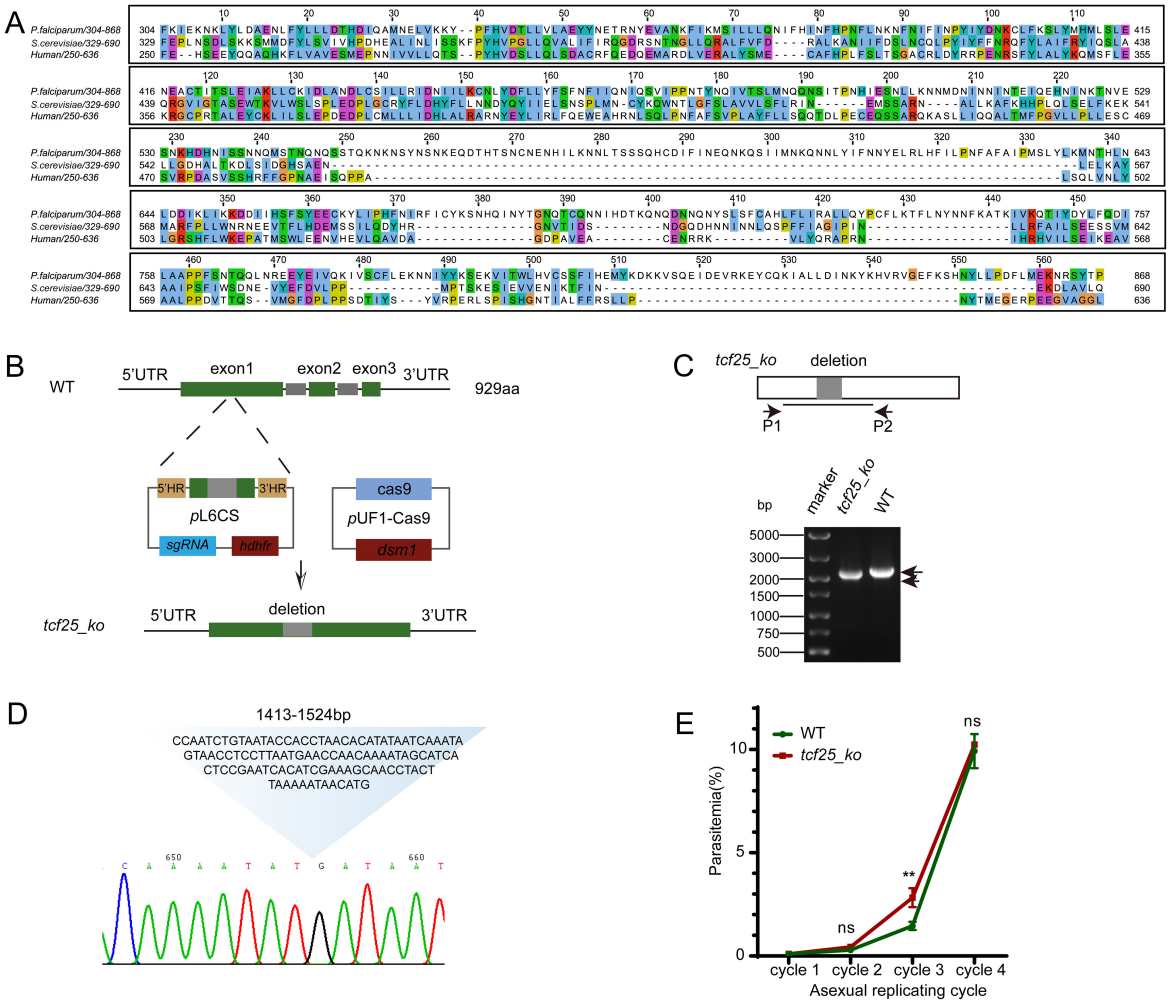
TCF25 deletion does not impair parasite replication during the asexual blood stage. **(A)** Alignment of PfTCF25 amino acid sequences from different species. The *tcf25* homologous genes were retrieved from *Saccharomyces cerevisiae* (QHB07799.1) and *Homo sapiens* (NP_055787.1). **(B)** Schematic diagram of CRISPR-mediated *tcf25* knockout in the WT strain. **(C)** DNA gel showing PCR validation of the *tcf25_ko* strain with the indicated primers. **(D)** Sanger sequencing results of the *tcf25_ko* strain, confirming the precise deletion of the 112bp target sequence. The chromatogram displays the edited genomic locus with the expected knockout junction. **(E)** Parasitemia dynamics during three consecutive culture cycles comparing WT and *tcf25_ko* strains. Data represent mean ± SEM (n=3 biological replicates). **p < 0.01; ns, not significant (two-tailed Student’s t-test).

The *P. falciparum* orthologue of TCF25, PF3D7_0506800, contains a coding region of 2,790 bp, which is composed of three exons and encodes a protein of approximately 110 kDa. In this study, we conducted a comprehensive multiple sequence alignment of the TCF25 functional domain sequences (304-868aa) from *P. falciparum* and two other diverse species: *Saccharomyces cerevisiae* and *Homo sapiens*. Our analysis revealed significant sequence conservation within the N-terminal region of the functional domain, suggesting strong evolutionary pressure to preserve this segment. However, compared to yeast Rqc1 and human Nulp1, PfTCF25 appears to have retained distinct evolutionary features, indicating that this protein may perform specialized functions in *Plasmodium* spp. (**Figure 1A**).

Previous studies have indicated that *tcf25* is a dispensable gene for asexual blood-stage development (Zhang et al., 2018). Therefore, we successfully deleted *tcf25* using a CRISPR/Cas9-based strategy. Given the considerable length of the *tcf25* gene, we selected a 112 bp region near the start codon as the target for deletion. The upstream and downstream DNA regions flanking the target sequence were PCR-amplified and cloned into the pL6CS vector, which has been previously employed for gene editing in *P. falciparum* (Shang et al., 2021). A 20 bp guide RNA sequence was also inserted into the vector. The resulting construct was co-transfected with the pUF1-Cas9 plasmid into WT parasites (**Figure 1B**). The deletion of the *tcf25* locus (*tcf25_ko*) parasite lines was confirmed by PCR and Sanger sequencing (**Figures 1C, D**). To assess the potential effect of *tcf25* deletion on asexual parasite replication, we conducted *in vitro* growth assays comparing WT and *tcf25_ko* strains over three replication cycles. Parasitemia was monitored by microscopic examination of Giemsa-stained thin blood smears. We observed a slight upregulation in the *tcf25_ko* strain during the third cycle. However, no significant difference was observed between WT and *tcf25_ko* strains in the fourth cycle (**Figure 1E**). These findings suggest that *tcf25* is dispensable for asexual blood-stage replication.

### *tcf25* deletion leads to genome-wide transcriptional alterations

**Figure 2:**
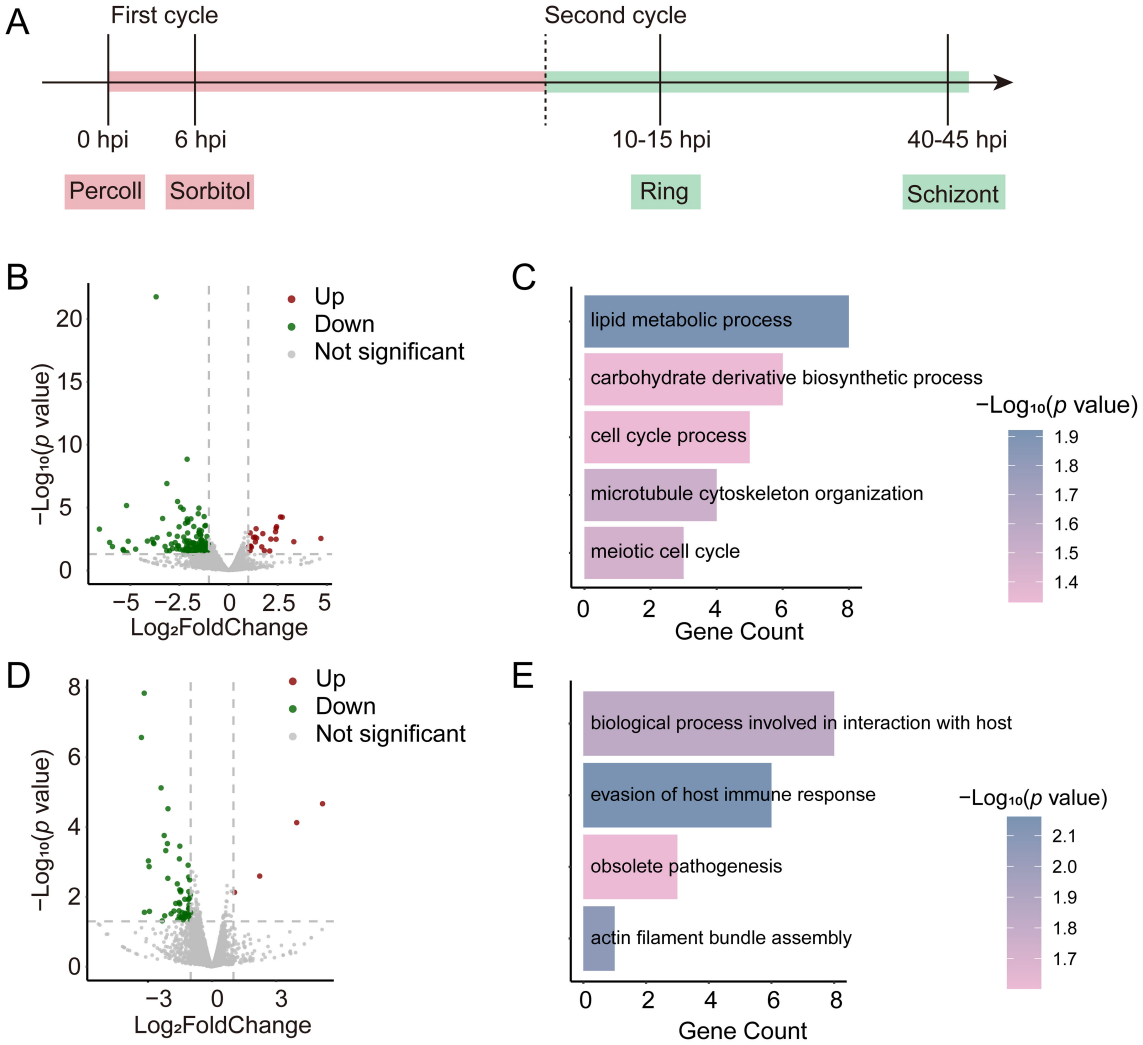
Transcriptional alterations in *tcf25_ko* versus WT strains. **(A)** Schematic of RNA-seq sample collection. Ring-stage and schizont-stage samples were harvested from tightly synchronized parasites during the second replication cycle (n=2 biological replicates). **(B)** Volcano plot displaying genome-wide differential gene expression analysis between *tcf25_ko* and WT strains at the ring stage. **(C)** GO enrichment analysis of biological processes associated with transcripts showing reduced abundance in *tcf25_ko* versus WT at the ring stage. **(D)** Volcano plot displaying genome-wide differential gene expression analysis between *tcf25_ko* and WT strains at the schizont stage. **(E)** GO terms for biological processes linked to transcripts with decreased abundance in *tcf25_ko* versus WT at the schizont stage.

Since the absence of *tcf25* does not affect the asexual replication in the blood stage, we further investigated its role in transcriptional regulation. RNA-seq was performed between WT and *tcf25_ko* parasite strains at the ring and schizont stages (**Figure 2A**). Comparative RNA-seq analysis of WT and *tcf25_ko* parasites identified 192 differentially expressed genes (DEGs) in the ring stage, with 24 upregulated and 168 downregulated in the knockout strain (**Figure 2B**; **Supplementary Tables S2**, **S3**). Upregulated genes were predominantly associated with antigenic variation, while Gene Ontology (GO) enrichment analysis of downregulated transcripts revealed significant enrichment for lipid metabolic process, carbohydrate derivative biosynthetic process, cell cycle process, microtubule cytoskeleton organization and meiotic cell cycle (**Figure 2C**). At the schizont stage, 57 DEGs were detected (4 upregulated, 53 downregulated) (**Figure 2D**; **Supplementary Tables S2**, **S3**). GO analysis linked these downregulated genes to biological processes involved in interaction with host, evasion of host immune response, obsolete pathogenesis, and actin filament bundle assembly (**Figure 2E**). These findings demonstrate that *tcf25* deletion exerts genome-wide transcriptional effects in *P. falciparum*.

### PfTCF25 is essential for gametocytogenesis

**Figure 3:**
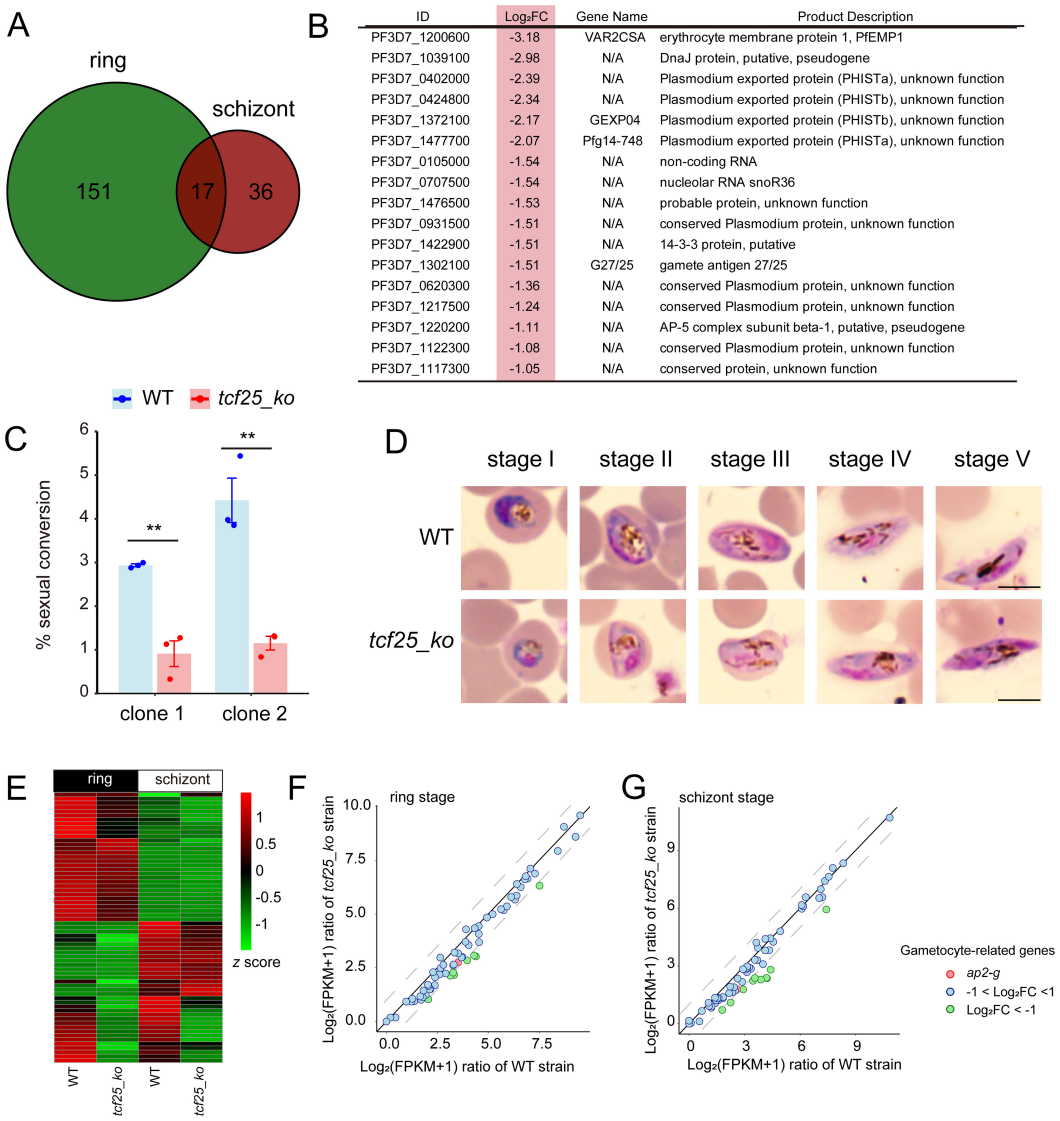
TCF25 deletion significantly affects gametocyte formation. **(A)** Venn diagram of downregulated transcripts at ring and schizont stages. **(B)** Commonly downregulated genes in both ring and schizont stages, with light red highlighting indicating their log_2_ fold changes at the schizont stage. **(C)** Sexual conversion rates of WT and two independently generated *tcf25_ko* clone strains following gametocyte induction (n=3 biological replicates). **(D)** Gametocyte development progression in WT and *tcf25_ko* strains. While *tcf25* deletion exhibited reduced gametocyte numbers, mature forms were still detectable. Scale bars: 5 μm. **(E)** Heatmap of expression changes for gametocyte-related genes in WT and *tcf25_ko* strains at the ring and schizont stage. **(F, G)** Scatter plot of expression changes for gametocyte-related genes in WT and *tcf25_ko* strains at the ring **(F)** or schizont **(G)** stage. **p < 0.01 (two-tailed Student’s t-test).

To explore consistently altered genes, we examined the intersection of downregulated DEGs after *tcf25* deletion at both the ring and schizont stages. The data revealed 17 consistently downregulated genes in the *tcf25_ko* strain, including *gexp04*, *pfg14-748*, and *g27/25*, which were associated with gametocyte development (**Figures 3A, B**) (Healer et al., 1997; Eksi et al., 2005). To evaluate the impact of *tcf25* deletion on sexual commitment and development, we induced gametocytogenesis in WT and *tcf25_ko* parasite lines and monitored developmental progression via morphological analysis of Giemsa-stained thin blood smears. To ensure reproducibility of the *tcf25* deletion phenotype, two independently generated *tcf25_ko* clones were used in this experiment. The *tcf25_ko* clone 1 line exhibited a marked reduction in gametocyte, with conversion efficiency decreased by approximately 69% compared to WT strain, while clone 2 displayed a reduction of approximately 74% in gametocyte production (**Figure 3C**). Nevertheless, gametocytes from the *tcf25_ko* strain displayed normal morphology and retained the ability to reach maturation, indicating that TCF25 is critical for efficient sexual commitment, while it is dispensable for gametocyte development (**Figure 3D**). To elucidate the molecular basis of this phenotype, we examined the transcriptional changes in gametocytogenesis-associated genes in *tcf25_ko* strain at the ring and schizont stages. Comparative expression analysis revealed a global downregulation of genes in sexual commitment and development (**Figure 3E**). Notably, *ap2-g*, the master transcriptional regulator of gametocytogenesis, was downregulated in the knockout line, although this reduction did not reach statistical significance (**Figures 3F, G**) (Kafsack et al., 2014). These findings demonstrate that *tcf25* deficiency disrupts the transcriptional activation of gametocyte conversion and development genes.

### TCF25 exhibits preferential localization to the gene bodies of target genes

**Figure 4:**
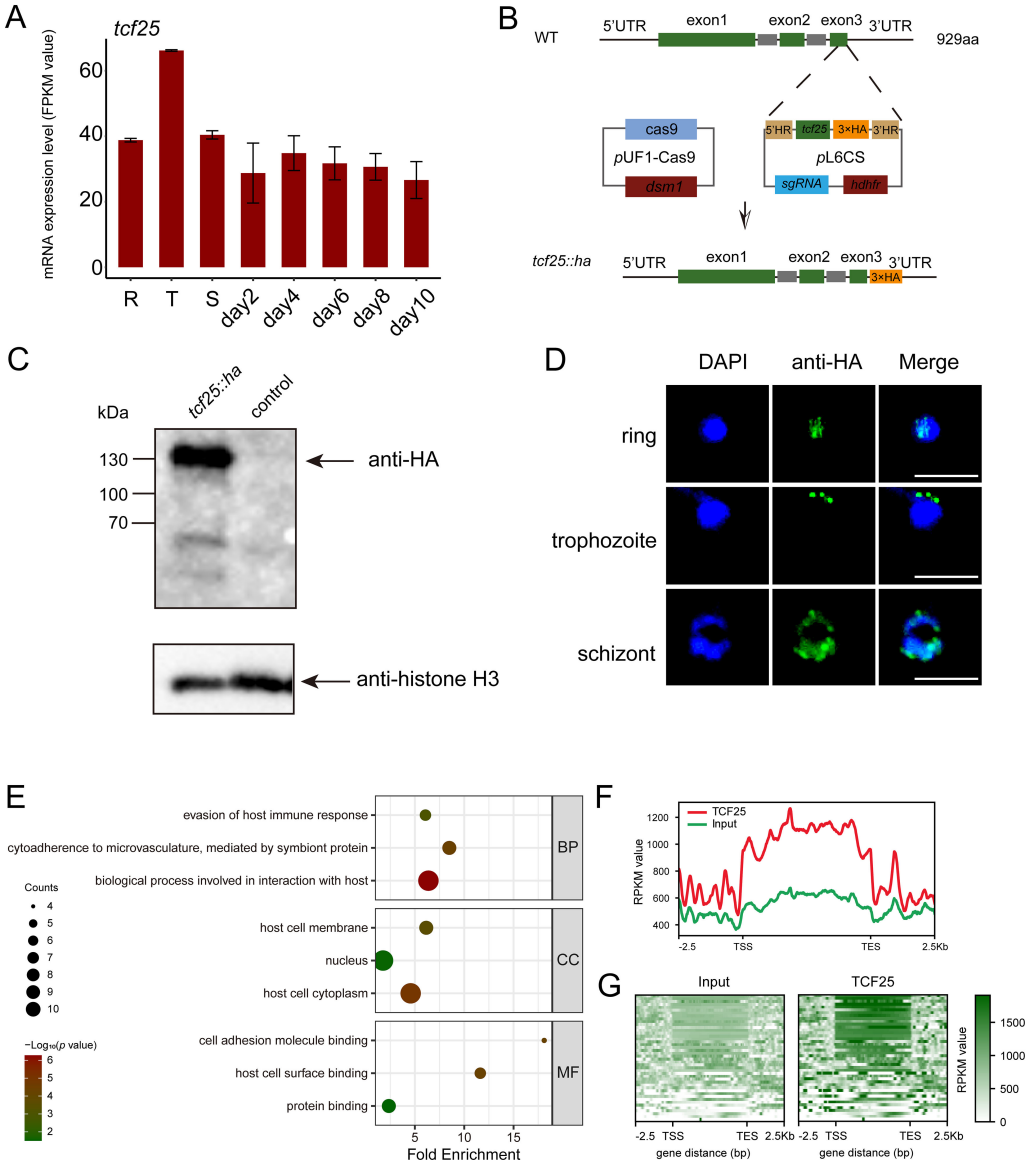
Identification of TCF25 protein-binding target genes. **(A)** Expression profile of *tcf25* during the IDC and gametocyte development (published dataset reanalysis). **(B)** Schematic of *tcf25* C-terminal HA-tagging strategy. **(C)** Western blot validation of TCF25-HA (expected molecular weight) with histone H3 loading control. **(D)** Localizations of TCF25 during asexual development stages. Representative IFA images showing HA signal (green), DNA (DAPI, blue), and merged channels at ring, trophozoite, and schizont stages. Scale bars: 5 μm. **(E)** GO enrichment analysis of TCF25 ChIP-seq targets (BP: biological processes, CC: cellular components, MF: molecular functions). **(F)** Line plot of TCF25 chromatin occupancy, showing average enrichment across all target genes. Profiles are aligned from 2.5 kb upstream of the transcription start site (TSS) to 2.5 kb downstream of the transcription end site (TES). **(G)** Heatmap of TCF25 chromatin occupancy, displaying enrichment across all target genes. Profiles are aligned from 2.5 kb upstream of the TSS to 2.5 kb downstream of the TES.

**Figure 5:**
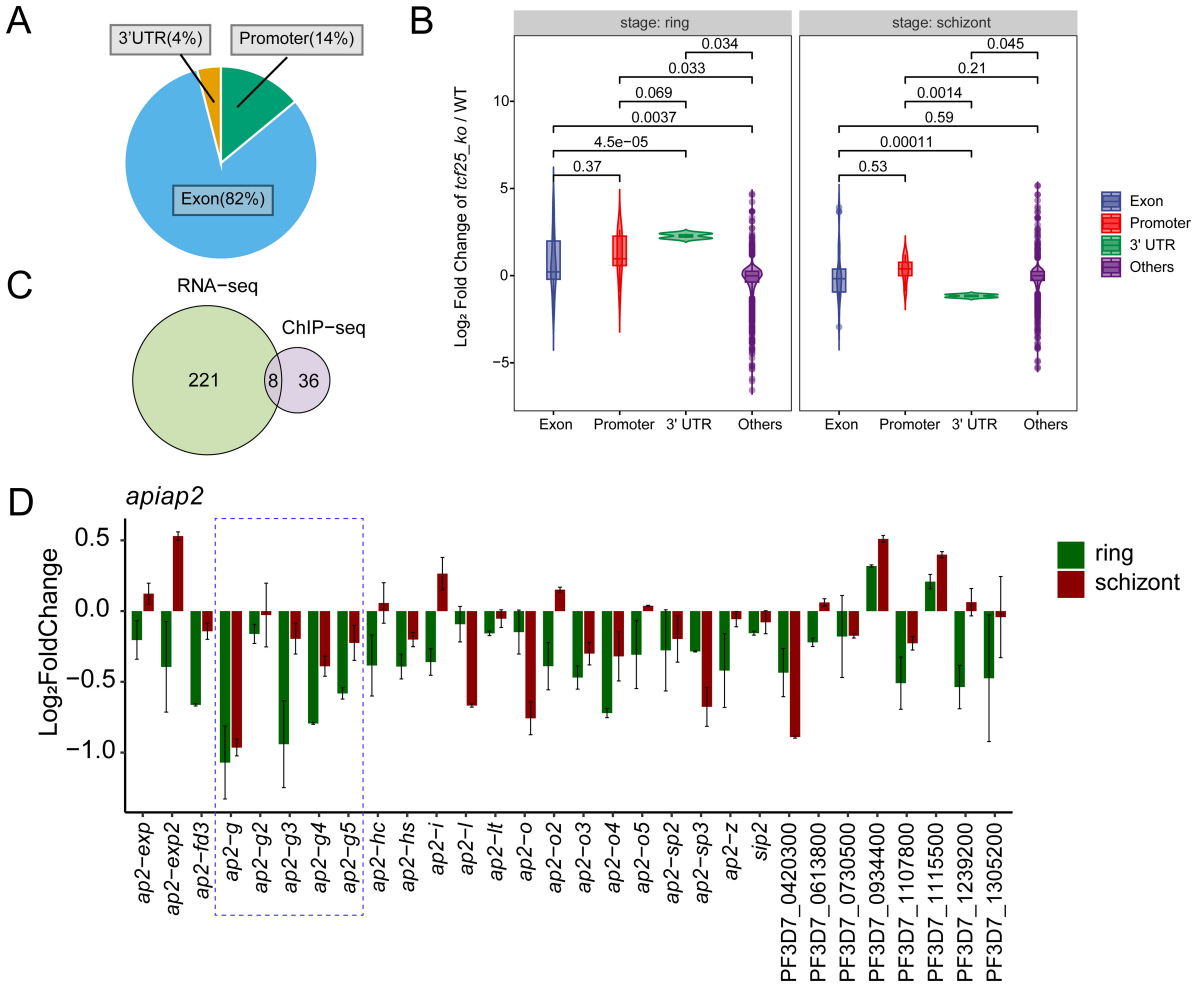
TCF25 influences gametocytogenesis via *ap2-g* pathway. **(A)** Pie chart showing the percentage of TCF25 peaks located in different genomic regions. **(B)** Violin plots display the distribution of gene expression changes (log_2_ fold change) for genes with TCF25 binding in different genomic regions. *P*-values indicating statistical significance of differences between groups are shown above each comparison. **(C)** Venn diagram comparing TCF25 binding genes and DEGs from transcriptomic analysis. **(D)** Expression changes (log_2_ fold change) of 30 ApiAP2 transcription factors in *tcf25_ko* versus WT.

To characterize the expression profile of *tcf25* across the intraerythrocytic development cycle, we analyzed recent transcriptomic data assessing mRNA abundance during asexual proliferation (R, T, and S) and gametocyte development (day2-10) (Shang et al., 2021). The data indicated that *tcf25* exhibits relatively stable expression throughout these stages, with only a modest elevation during the trophozoite stage (**Figure 4A**). This suggests a potential functional role for TCF25 in both asexual and sexual stages. To determine the subcellular localization of TCF25, we generated a *tcf25::ha*-tagged parasite line (**Figure 4B**). HA-tagged TCF25 protein was successfully detected by Western blot analysis (**Figure 4C**). Immunofluorescence assay (IFA) confirmed that TCF25 is expressed throughout asexual blood stages, with nuclear localization and cytoplasmic distribution (**Figure 4D**). This nuclear enrichment suggests TCF25 may function as a nuclear regulatory factor. To systematically identify TCF25’s molecular targets, we performed ChIP-seq using synchronized schizont-stage parasites. Peak calling analysis revealed 44 high-confidence TCF25 binding genes distributed across the parasite genome (**Supplementary Tables S4**, **S5**). GO analysis revealed that these target genes are associated with biological process involved in interaction with host, cytoadherence to microvasculature, mediated by symbiont protein, evasion of host immune response, *etc.* (**Figure 4E**). Genomic mapping identified significant TCF25 enrichment in gene bodies, with additional occupancy detected at promoter regions and 3’ UTRs (**Figures 4F, G**). We next categorized TCF25-bound peaks by genomic distribution, revealing occupancy across diverse regulatory elements: promoter (14%), exon (82%), and 3’ UTR (4%) (**Figure 5A**). Integrated analysis of ChIP-seq and transcriptomic data demonstrated that during the ring stage, TCF25-bound genes showed significantly upregulated expression relative to non-bound genes (*p* < 0.05). This regulatory pattern was absent during the schizont stage (**Figure 5B**). These results imply that TCF25 likely acts as a transcriptional repressor.

### TCF25 contributes to gametocytogenesis through the *ap2-g* pathway

To investigate the role of TCF25 in gene expression, we integrated ChIP-seq data with transcriptomic profiles from WT and *tcf25* knockout parasites. Intersection analysis between TCF25-bound target genes and DEGs from transcriptomic data identified 8 overlapping genes (**Figure 5C**). This subset was predominantly composed of rRNA genes. Considering the well-established regulatory functions of ApiAP2 transcription factors in orchestrating parasite stage transitions, we performed comprehensive transcriptional profiling to characterize expression alterations in ApiAP2 genes upon *tcf25* knockout. The analysis of all 30 ApiAP2 transcription factors revealed stage-specific dysregulation: 93% (28/30) demonstrated reduced expression during the ring stage, while 63% (19/30) were downregulated in schizont stages (Singhal et al., 2024). The most pronounced effects occurred within the *ap2-g* subfamily, with *ap2-g, ap2-g3*, and *ap2-g4* showing the greatest suppression, consistent with their known functions in sexual commitment (**Figure 5D**). Together, these results demonstrate that TCF25 mediates gametocytogenesis through the *ap2-g* pathway.

### TCF25 is essential in ribosome biogenesis

**Figure 6:**
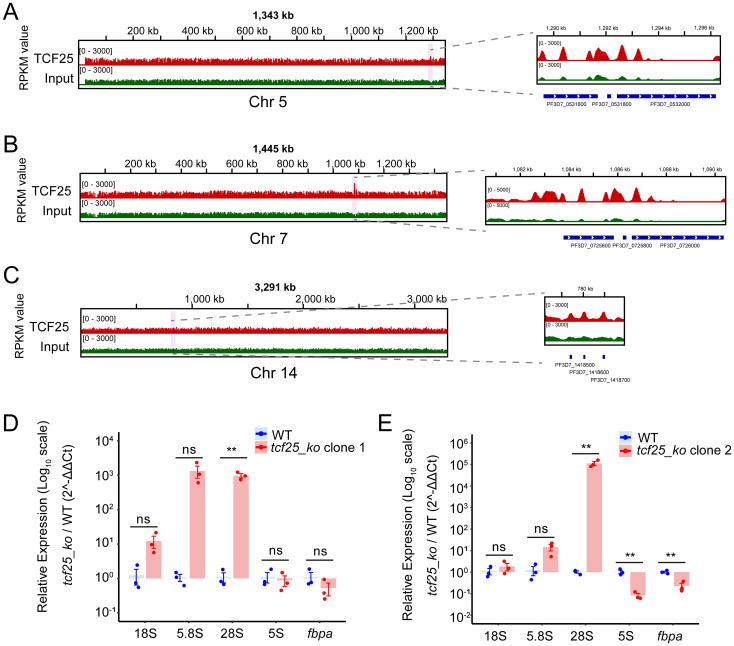
TCF25 significantly binds and regulates rDNA. **(A–C)** IGV visualization of TCF25 protein distribution on chromosome 5 **(A)**, chromosome 7 **(B)**, and chromosome 14 **(C)**. The left panel shows the distribution across the chromosome, while the right panel displays the distribution on the rDNA clusters. The light pink highlighted regions indicate the chromosomal locations of the zoomed-in areas. **(D, E)** Quantitative RT-qPCR analysis of rRNA expression in two independently generated *tcf25_ko* clone lines. Data represent mean ± SEM of three biological replicates, normalized to PF3D7_0717700 and expressed relative to WT expression levels. Relative expression levels of the *fbpa* gene were used as positive controls. **p < 0.01; ns, not significant (two-tailed Student’s t-test).

As a member of the RQC complex, TCF25 is critically involved in ribosomal rescue pathways by facilitating the disassembly and release of stalled 60S subunits from arrested ribosomes, thereby maintaining translational fidelity. In *P. falciparum*, four distinct rRNA species (28S, 5.8S, 5S, and 18S rRNA) are encoded as gene clusters distributed across chromosomes 1, 5, 7, 8, 11, 13, and 14. These rRNAs can be classified into two major types: A-type and S-type. The A-type rRNAs (A1 and A2) are expressed during the asexual blood stage, whereas the S-type rRNAs (S1 and S2) are active in the gametocyte and sporozoite stages (Fang et al., 2004). Genome-wide binding profiling revealed significant enrichment of TCF25 specifically at rDNA clusters on chromosomes 5, 7, and 14, which primarily encode A-type rRNAs. IGV visualization demonstrated predominant localization within coding regions, along with peaks at promoter elements. Chromosome 7 exhibited the strongest TCF25 binding signal among all rDNA loci (**Figures 6A–C**). Given the limitations of RNA-seq in accurately quantifying rRNA levels, we performed RT-qPCR to measure rRNA abundance in two independent *tcf25_ko* clones. Notably, both clones exhibited a significant upregulation of 28S rRNA transcripts (*p* < 0.01), a core structural component of the 60S ribosomal subunit (**Figures 6D, E**). This finding suggests that TCF25 deficiency may impair the recycling of 60S subunits, leading to their compensatory overproduction. Collectively, these results suggest the critical role of TCF25 in ribosome biogenesis and ribosomal subunit homeostasis.

## Discussion

The proliferation of *Plasmodium* spp. occurs through asexual replication in erythrocytes, leading to pathogenesis via cell destruction. However, a small subset of sexually committed parasites undergo a lengthy developmental process to differentiate into mature gametocytes for transmission. In this study, we investigated the bHLH transcription factor TCF25 and found that genetic deletion of *tcf25* significantly reduces sexual conversion efficiency. This discovery highlights TCF25 as a previously unrecognized regulatory factor in the sexual development pathway, enhancing our understanding of the mechanisms involved in this crucial biological transition essential for malaria transmission.

Our extensive analysis of TCF25 by targeted gene disruption unveils its diverse involvement in the regulation of *P. falciparum* development. Specifically, CRISPR/Cas9-mediated deletion of nucleotides 1413–1524 resulted in loss of protein integrity without impacting asexual proliferation. Transcriptomic profiling illustrated TCF25’s widespread regulatory impact, with a notable decrease in the expression of genes associated with gametocytes (such as *gexp04, pfg14-748*, and *g27/25*) during both the ring and schizont stages. This was further supported by a marked reduction in the sexual conversion rate of knockout parasites. Genome-wide mapping of TCF25 occupancy identified 44 direct targets. Additionally, the unexpected identification of TCF25’s strong association with rDNA loci indicates a potential conserved role in ribosome biogenesis, highlighting this bHLH-family factor as a multifaceted coordinator that links developmental transitions with translational control in malaria parasites.

**Figure 7:**
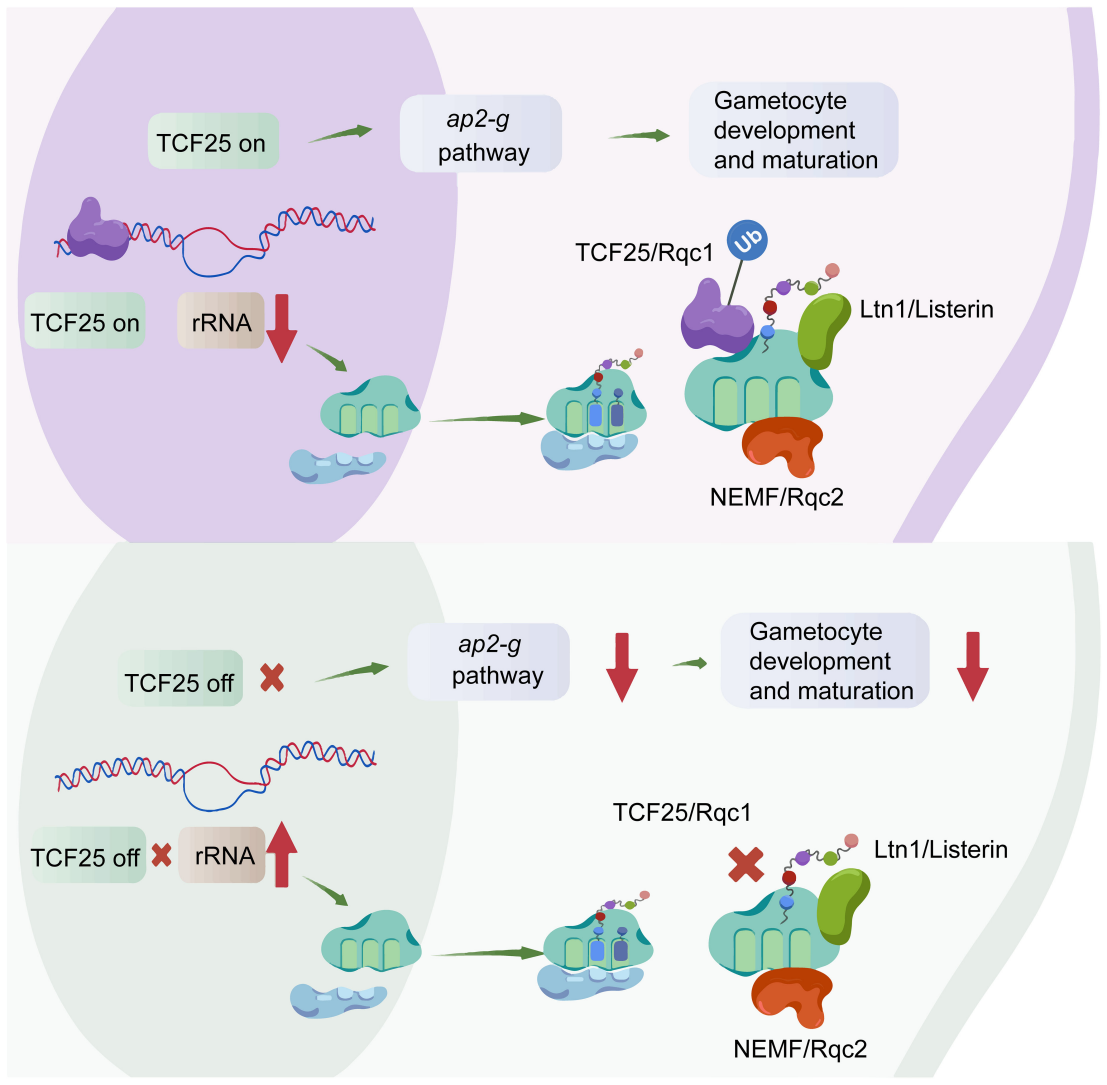
Model of TCF25’s dual regulation of gametocytogenesis and ribosome biogenesis. Created with BioGDP (https://biogdp.com/).

In other eukaryotes, TCF25 is a key component of the RQC complex, along with the ubiquitin ligase Ltn1(Listerin) and Rqc2 (NEMF), responsible for releasing stalled ribosomes’ 60S subunit and directing nascent polypeptides for degradation (Brandman et al., 2012; Filbeck et al., 2022). However, the role of TCF25 in this process remains incompletely understood. Besides its role in RQC-mediated ubiquitination, there is growing evidence suggesting that TCF25 may also have evolutionarily conserved transcriptional regulatory functions. Eukaryotic ribosomes, comprising 40S and 60S subunits, perform distinct yet coordinated roles in protein synthesis: the 40S subunit, containing 18S rRNA and ribosomal proteins, facilitates mRNA binding, decoding, and initiation, while the 60S subunit, composed of 28S, 5.8S, and 5S rRNAs and ribosomal proteins, catalyzes peptide bond formation and tRNA translocation. Our results demonstrate that TCF25 is significantly enriched at A-type rRNAs, and its depletion leads to marked upregulation of rRNAs, particularly 28S rRNA. This could reflect either a direct repressive role of TCF25 in rRNA transcription or defective recycling of stalled 60S subunits, prompting compensatory ribosome production. These findings strongly suggest that TCF25 acts as a suppressor of ribosome biogenesis, potentially through direct regulation of rDNA loci or indirect modulation of ribosome assembly pathways (**Figure 7**).

In conclusion, our comprehensive multi-omics analysis has unveiled novel functions of TCF25 in the regulation of malaria parasite transmission and gene expression, shedding new light on gametocytogenesis and ribosome biogenesis in *P. falciparum*. While these discoveries enhance our comprehension of parasite biology, it is essential to address several key limitations. The current investigation predominantly focused on the blood stage, leaving unanswered queries regarding TCF25’s potential involvement in gametogenesis and other crucial lifecycle transitions. While gene knockout is a valuable tool for revealing phenotypic outcomes, its inability to conditionally control TCF25 expression limits its utility for investigating the gene’s stage-specific functions. Consequently, the development of an effective inducible knockdown system is needed to explore the roles of TCF25. Furthermore, a more thorough validation is necessary to ascertain the composition and functional arrangement of the proposed RQC complex in *P. falciparum*. Moving forward, our research will concentrate on elucidating the precise molecular interactions between TCF25 and other RQC constituents, along with delineating its mechanistic contributions to RQC, including its possible participation in the ubiquitin-dependent degradation of faulty nascent chains. These forthcoming research endeavors not only aim to refine our understanding of TCF25’s diverse functions but also hold the promise of unveiling novel facets of translational regulation in malaria parasites.

## Data Availability

The sequenced raw data have been deposited in the National Center for Biotechnology Information (NCBI) Gene Expression Omnibus (GEO) with the accession numbers GSE298804 and GSE298805. The datasets presented in this study can be found in online repositories.
